# A Fast, Reliable Oil-In-Water Microemulsion Procedure for Silica Coating of Ferromagnetic Zn Ferrite Nanoparticles Capable of Inducing Cancer Cell Death In Vitro

**DOI:** 10.3390/biomedicines10071647

**Published:** 2022-07-08

**Authors:** Stefan Nitica, Ionel Fizesan, Roxana Dudric, Lucian Barbu-Tudoran, Anca Pop, Felicia Loghin, Nicoleta Vedeanu, Constantin Mihai Lucaciu, Cristian Iacovita

**Affiliations:** 1 Department of Pharmaceutical Physics-Biophysics, Faculty of Pharmacy, “Iuliu Hațieganu” University of Medicine and Pharmacy, 6 Pasteur St., 400349 Cluj-Napoca, Romania; stefan_nitica@yahoo.com (S.N.); nicoletavedeanu@yahoo.com (N.V.); 2 Department of Toxicology, Faculty of Pharmacy, “Iuliu Hațieganu” University of Medicine and Pharmacy, 6A Pasteur St., 400349 Cluj-Napoca, Romania; ionel.fizesan@umfcluj.ro (I.F.); anca.pop@umfcluj.ro (A.P.); floghin@umfcluj.ro (F.L.); 3 Faculty of Physics, “Babes-Bolyai” University, Kogalniceanu 1, 400084 Cluj-Napoca, Romania; roxana.dudric@ubbcluj.ro; 4 Electron Microscopy Center “Prof. C. Craciun”, Faculty of Biology & Geology, “Babes-Bolyai” University, 5–7 Clinicilor St., 400006 Cluj-Napoca, Romania; lucian.barbu@ubbcluj.ro; 5 Electron Microscopy Integrated Laboratory, National Institute for Research and Development of Isotopic and Molecular Technologies, 67–103 Donath St., 400293 Cluj-Napoca, Romania

**Keywords:** zinc ferrite nanoparticles, silica coating, oil-in-water microemulsion, ultrasonication, magnetic hyperthermia, cancer cells, A549 cells, BJ cells, Alamar Blue, Neutral Red

## Abstract

The applications of ferrimagnetic nanoparticles (F-MNPs) in magnetic hyperthermia (MH) are restricted by their stabilization in microscale aggregates due to magnetostatic interactions significantly reducing their heating performances. Coating the F-MNPs in a silica layer is expected to significantly reduce the magnetostatic interactions, thereby increasing their heating ability. A new fast, facile, and eco-friendly oil-in-water microemulsion-based method was used for coating Zn_0__.__4_Fe_2.6_O_4_ F-MNPs in a silica layer within 30 min by using ultrasounds. The silica-coated clusters were characterized by various physicochemical techniques and MH, while cytotoxicity studies, cellular uptake determination, and in vitro MH experiments were performed on normal and malignant cell lines. The average hydrodynamic diameter of silica-coated clusters was approximately 145 nm, displaying a high heating performance (up to 2600 W/g_Fe_). Biocompatibility up to 250 μg/cm^2^ (0.8 mg/mL) was recorded by Alamar Blue and Neutral Red assays. The silica-coating increases the cellular uptake of Zn_0.4_Fe_2.6_O_4_ clusters up to three times and significantly improves their intracellular MH performances. A 90% drop in cellular viability was recorded after 30 min of MH treatment (20 kA/m, 355 kHz) for a dosage level of 62.5 μg/cm^2^ (0.2 mg/mL), while normal cells were more resilient to MH treatment.

## 1. Introduction

In the field of novel cancer therapy, magnetic nanoparticles (MNPs) are widely investigated within magnetic hyperthermia (MH) treatment [1,2,3]. The technique exploits the Néel magnetic relaxation, Brownian rotation, and hysteresis losses of MNPs, when subjected to an alternating magnetic field (AFM), to generate sufficient heat for inducing cancer cell death [4,5,6]. Ferri- or ferromagnetic nanoparticles (F-MNPs) are most desirable for MH applications since their heating capabilities are one order of magnitude greater in comparison with superparamagnetic iron oxide nanoparticles (SPIONs), due to the increase in both their size and dynamic hysteresis area [7,8,9]. However, the F-MNPs present a great disadvantage for in vivo and in vitro MH applications as they possess poor colloidal stability in biological media. The permanent magnetic moments of F-MNPs give rise to magnetostatic interactions among them that hinder their dispersion in biological media and favor their stabilization in microscale aggregates [10,11]. The magnetostatic interactions among F-MNPs depend on the distances between them, therefore these interactions may be reduced by introducing a non-magnetic coating around F-NPMag [12,13].

One of the most used methods for the surface modification of MNPs is silica coating [14]. The silica layer around F-MNPs avoids their interaction and agglomeration, providing colloidal stability and keeping them as effective magneto-mechanical actuators required for MH applications [15,16,17,18,19]. On the other hand, the intrinsic toxicity of MNPs is significantly reduced under the protection of silica shells, which have been demonstrated to be nontoxic and biocompatible for biomedical research [20,21,22,23,24]. The silica shells are visible and transparent to near-infrared, and the ultraviolet regions do not interfere with magnetic fields, allowing the MNPs to keep their original magnetic properties [25,26,27,28,29]. Moreover, the silica chemistry facilitates the easy attachment of functional molecules—especially those bearing amine of carboxyl groups—thus adding new functionalities to the hybrid nanostructures, such as fluorescence [29,30,31] and therapeutic [31,32,33] and catalytic functions [34]. As it was recently demonstrated, the attachment of a chemotherapeutic agent to the MNPs, reduced the cytotoxic effect of the drug in non-cancerous cells, improved the internalization in cancer cells, and its activity was synergistically enhanced in combination with magnetic hyperthermia [35]. Thus, the controlled encapsulation of the MNPs in a silica shell, by preserving their magnetic properties, enhancing their biocompatibility, and increasing their ability for functionalization with various functional groups represents a huge potential for future clinical translation of the MH technique.

The encapsulation of pre-synthesized MNPs in a SiO_2_ layer was usually performed through the hydrolysis of tetraethyl orthosilicate (TEOS) by an aqueous solution of ammonia -NH_3(aq)_ and the subsequent condensation of silica precursors on the surface of the MNPs. A well-known procedure is based on heterogeneous mixtures of water and an organic solvent (usually cyclohexane) formed and stabilized with the help of a surfactant (a widely used one is polyoxyethylene nonyl phenyl ether-Igepal CO-520) called water-in-oil (*w*/*o*) microemulsions. The nanometer-sized water droplets dispersed in the organic solvent act as nanoreactors capable of forming a SiO_2_ layer around MNPs under a mechanical stirring over at least 12 h [19,20,21,23,26,27,30,31,34,36,37,38,39,40]. This method was mainly applied to SPION_S,_ and it allowed the formation of a homogeneous SiO_2_ layer and fine control of its thickness, around individual SPIONs. Recently, our research group succeeded in encapsulating polyhedral F-NPMag clusters following the *w*/*o* microemulsion method [41]. Another commonly used method for encapsulating MNPs in a SiO_2_ layer was based on the Stöber process in a sol-gel approach in which the organic solvent was replaced by ethanol or propanol [15,16,17,18,22,24,25,29,33,42,43,44]. This method requires an additional step in which the pre-synthesized MNPs are coated with a polymer, usually polyvinylpyrrolidone (PVP), to make their encapsulation in the SiO_2_ layer more efficient.

The main disadvantage of both methods is the very long duration (from a few hours to a few days) necessary to form the SiO_2_ layer around MNPs. The use of SiO_2_-coated MNPs in both in vitro and in vivo studies, and later in clinical trials, requires large quantities of MNPs@SiO_2_, the preparation of which can last even weeks/months due to the long duration of the synthetic procedure. Therefore, the elaboration of a procedure by which the SiO_2_ coating of any type of MNPs can be achieved as quickly and efficiently as possible is a major purpose in this field. Moreover, in the case of F-MNPs, the encapsulation procedure must also inhibit the formation of large (micrometric) aggregates before the growth of the SiO_2_ layer around the pre-existing F-MNPs clusters following the synthesis method.

Herein, we report the coating of ferromagnetic zinc (Zn) doped iron oxide nanoparticles with an SiO_2_ layer by using oil-in-water (*o*/*w*) microemulsions, which eliminates the need for the expensive surfactant Igepal CO-520 and for large volumes of organic solvent (cyclohexane) in the synthesis process, shortening the overall time frame to a maximum of 30 min. The magnetic cores and the coatings were fully characterized using multiple analytical methods such as transmission electron microscopy (TEM), infrared spectroscopy (FT-IR), X-ray diffraction (XRD), and dynamic light scattering (DLS). Further, the magnetic and the heating properties were analyzed by a vibrating sample magnetometer (VSM) and a magnetic hyperthermia (MH) system. The biological assessment included cytotoxicity and cellular uptake determination in cancerous and normal cell lines. Finally, we demonstrated the potential of the silica-coated clusters to induce cell death in vitro.

## 2. Materials and Methods

### 2.1. Synthesis of MNPs

The Zn ferrites were synthesized through thermal decomposition of a mixture of metal acetylacetonates with surfactants in a high-boiling point organic solvent. Briefly, iron (III) acetylacetonate (1.5 mmol) (Merck Schuchardt OHG, Hohenbrunn, Germany), zinc (II) acetylacetonate (2.25 mmol) (Merck KGaA, Darmstadt, Germany), oleic acid (3.8 mmol) (Sigma-Aldrich, Steinheim, Germany), and 10 mL benzyl ether (Sigma-Aldrich, Steinheim, Germany) were mixed and magnetically stirred under a flow of nitrogen. The mixture was placed in a 50 mL three-neck, round-bottom flask and heated to 300 °C, at a ramping rate of 5 °C/min, under a continuous flow of nitrogen. The mixture was kept at reflux for 30 min and then cooled down to room temperature by removing the heat source. The Zn ferrites were separated by a neodymium magnet and washed in a mixture of hexane (Honeywell, Seelze, Germany)–ethanol (Chemical, Iasi, Romania) (1:1, *v*/*v*) five times. Finally, the as-synthesized Zn ferrites were dispersed in a volume of hexane at a concentration of 10 mg_MNPs_/mL and stored in a glass container.

### 2.2. Silica Coating of MNPs

The silica shell was coated on the hydrophobic Zn ferrites via the microemulsion method. First, we tested the water-in-oil emulsion method, which was previously reported in the literature. We tried to improve this method by using ultrasounds for reducing the preparation time. Afterward, because the size of the clusters was too large, we tested a new method based on the oil-in-water emulsion. The emulsions were prepared in 100 mL glass bottles with a thread and a cap, using the following recipes:

- Classical water-in-oil microemulsion procedure: Briefly, 18 mL cyclohexane (Sigma-Aldrich, Steinheim, Germany) and 1.2 mL Igepal CO-520 (Sigma-Aldrich, Steinheim, Germany) were mixed for 30 min. Afterward, 4 mg of Zn_0.4_Fe_2.6_O_4_ dispersed in 2 mL cyclohexane were added, while stirring. After 5 min, 0.05 mL APTES (Sigma-Aldrich, Steinheim, Germany) and 0.1 mL TEOS (Sigma-Aldrich, Steinheim, Germany) were added, followed by 0.15 mL aqueous ammonia solution (25%). The dispersions were stirred at room temperature for 24 h.
- Ultrasound assisted water-in-oil microemulsion procedure: Briefly, 18 mL cyclohexane, 1.2 mL Igepal CO-520, 0.4 mL colloidal suspension in hexane (containing 4 mg of Zn_0.4_Fe_2.6_O_4_ NPs), 0.1 mL TEOS, and 0.05 mL APTES were mixed for 30 min, following the addition of 0.5 mL 25% aqueous ammonia solution and 1.5 mL ultrapure water. The mixture was subjected to ultrasonication for 30 min in a water bath sonicator Elmasonic S 30 (Elma Schmidbauer GmbH, Singen, Germany) operating at 37 kHz, with an effective acoustic power of 80 W in continuous mode.
- Ultrasound-assisted oil-in-water microemulsion procedure: Briefly, 18 mL of ultrapure water, 0.8 mL of TWEEN 20 (Sigma-Aldrich, Steinheim, Germany), 4 mg of Zn ferrites dispersed in 0.4 mL of hexane, and 0.1 mL of TEOS were introduced in a bottle with cap. The mixture was mixed for 5 min by alternating ultrasonication with short cycles (approx. 5 s) of manual stirring, after which 2 mL of 25% (*m*/*v*) aqueous ammonia solution was added, and the mixture was subjected to ultrasonication for 30 min. In the last 3 min of sonication, the bottle cap was removed to facilitate the evaporation of the solvent from the fine droplets and the complete encapsulation of the remaining MNPs.

The mixtures were further treated with an equal volume of ethanol and the silica-coated Zn ferrites were separated by a neodymium magnet. The Zn_0.4_Fe_2.6_O_4_@SiO_2_ were furthermore washed in ethanol once and in water three times before being redispersed in water (2 mg MNPs core per mL of water) for storage.

### 2.3. Characterization Methods

The morphology and the chemical composition of nanostructures were studied by transmission electron microscopy (TEM), using a Hitachi HD2700 (Hitachi, Tokyo, Japan) microscope operating at 200 kV and coupled with an EDX (energy-dispersive X-ray) detector (Oxford Instruments, Oxford, UK, AZtec Software, version 3.3), employing carbon-coated copper grids. Powder X-ray diffraction (XRD) measurements were performed with a Bruker D8 Advance diffractometer using Cu Kα radiation (Bruker AXS GmbH, Karlsruhe, Germany). Fourier transform infrared (FT-IR) spectra were recorded on a TENSOR II instrument (Bruker Optics Inc., Billerica, MA, USA) in attenuated total reflectance mode, using the platinum attenuated total reflectance (ATR) accessory with a single reflection diamond ATR. The spectra were recorded with a resolution of 4 cm^−1^ and 16 scans per sample between 400 and 4000 cm^−1^. Particle solutions with a concentration of 10 μg_MNPs_/mL were employed to determine the hydrodynamic size and zeta potential using a Zetasizer Nano ZS90 (Malvern Instruments, Worcestershire, UK) operating at room temperature in a 90° configuration. Magnetization measurements were carried out using a Cryogenic Limited (London, UK) vibrating sample magnetometer (VSM) under applied magnetic fields from 0 to ±4 T at both 4 K and 300 K. The heating efficiency was evaluated using a commercially available magnetic hyperthermia system, the Easy Heat 0224 from Ambrell (Scottsville, NY, USA) equipped with an optical fiber temperature sensor (0.1 °C accuracy). A volume of 0.5 mL of MNPs was placed in the center of an 8-turn coil using a thermally isolated Teflon holder and then submitted to an AC magnetic field with fixed frequency (355 kHz) and variable amplitude (5–65 kA/m). Details of specific absorption rate (SAR) calculations are provided in the Supplementary Materials (Section S1).

### 2.4. Cell Lines

For in vitro studies, a human pulmonary carcinoma A549 cell line and a human foreskin fibroblast BJ cell line were purchased from American Type Culture Collection (ATCC, Manassas, VA, USA) and used. Cells were maintained in Dulbecco’s Modified Eagle Medium (DMEM, Gibco, Paisley, UK) supplemented with 10% Fetal Bovine Serum (FBS, Gibco, Paisley, UK) at a temperature of 37 °C and 5% CO_2_ supplementation. Media was changed every other day, and the cellular cultures were either sub-cultured or used in the experiments at a confluency of 80–90%.

### 2.5. In Vitro Cytocompatibility Assays

Alamar Blue (AB) and Neutral Red (NR) assays were used to evaluate the cytotoxicity upon a 24 h exposure of the two cell lines to both Zn_0.4_Fe_2.6_O_4_@SiO_2_ and Zn_0.4_Fe_2.6_O_4_ NPs. Both types of cells were seeded in 6-well plates for 24 h, following 24 h exposure to NPs suspension at reached doses of 16, 31, 62.5, 125, and 250 µg/cm^2^. The AB and the NR dyes were then added upon thoroughly washing the cells with PBS. A volume of 200 µM resazurin solution was added; and, cells were incubated for 3 h, while the fluorescence was measured at an λ_excitation_ = 530/25 nm and λ_emission_ = 590/35 nm for the AB assay. A filtered NR dye solution (40 μg/mL) was used to incubate cells for 2 h, following a washing step to remove the non-internalized dye for NR assay. A 50% hydroalcoholic solution containing 1% glacial acetic acid was further employed to solubilize the intra-cellular accumulated dye, while the fluorescence was measured at a λ_excitation_ = 530/25 nm and a λ_emission_ = 620/40 nm. A Synergy 2 Multi-Mode Microplate Reader was used for fluorescence measurements that were realized in biological triplicates.

### 2.6. Evaluation of Cellular Uptake

The cellular uptake of both Zn_0.4_Fe_2.6_O_4_@SiO_2_ and Zn_0.4_Fe_2.6_O_4_ NPs was evaluated upon 24 h incubation. Qualitative evaluation was performed using Prussian Blue staining: cells were fixed with 4% paraformaldehyde for 30 min, and stained with a mixture containing a 2% HCl and a 2% potassium ferrocyanide aqueous solutions; after the development of the blue color, cells were washed three times with PBS, counterstained with Eosin, and finally visualized under a light microscope at a magnification of 100×. For quantitative determinations, the cells were washed, trypsinized, centrifuged for 5 min at 4500× *g*, and then processed for the Fe^3+^ quantification using the Liebig reaction of free Fe^3+^ with thiocyanate, as described in the Supplementary Materials (Section S2).

### 2.7. In Vitro Magnetic Hyperthermia

A549 and BJ cells were seeded in 6-well plates for 24 h and then further exposed for an additional 24 h to Zn_0.4_Fe_2.6_O_4_@SiO_2_ NPs to reach the following concentrations: 31, 62.5, and 125 µg/cm^2^. After the exposure, cells were thoroughly washed with PBS, detached using 300 µL of trypsin (0.05%) and then further neutralized with 2700 µL media containing FBS. The cellular suspension was equally divided into two aliquots, centrifuged for 10 min at 100× *g*, and 1300 µL of supernatant was removed from both aliquots. One of the aliquots was kept in a water bath at 37 °C (negative control), while the other aliquot was exposed to an alternating magnetic field (AMF) for 30 min, working at a fixed frequency of 355 kHz and amplitudes of 15, 20, and 30 kA/m. The cells were placed in an Eppendorf tube in the middle of an 8-turn coil. The tube was surrounded by plastic pipes connected to a Peltier element and thermostated at 37 °C. After the AMF exposure, cells from exposed and unexposed aliquots were plated in 96-wells as 6 technical replicates, and cellular viability was measured using the AB and the NR assays after 24 h. Each experiment was performed in three biological replicates. Data were normalized to the AMF negative control (cells exposed to Zn_0.4_Fe_2.6_O_4_@SiO_2_ NPs but not to the AMF).

### 2.8. Statistics

The data are presented as average values ± standard deviation (SD). Data sets were analyzed using a One-way Analysis of Variance (ANOVA) with a post-hoc + Dunn’s test, and the graphical representations were done in SigmaPlot 11.0 computer software (Systat). Statistical results showing *p* values < 0.05 were considered statistically significant.

## 3. Results and Discussion

### 3.1. Magnetic Nanoparticles Characterization

**Figure 1:**
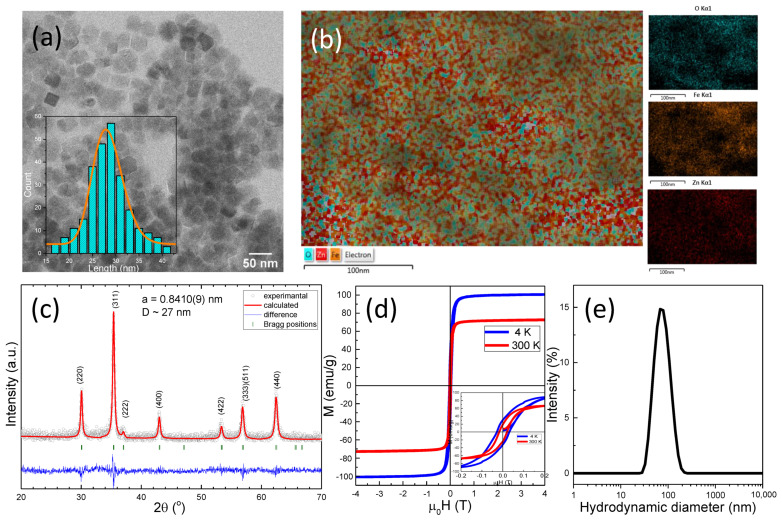
(**a**) TEM images of Zn_0.4_Fe_2.6_O_4_ NPs. Inset represents the size distribution histogram of Zn_0.4_Fe_2.6_O_4_ NPs fitted to a log-normal distribution (orange line). (**b**) EDX global chemical map together with chemical maps of Fe, Zn, and O elements of Zn_0.4_Fe_2.6_O_4_ NPs. (**c**) XRD diffraction pattern of Zn_0.4_Fe_2.6_O_4_ NPs. (**d**) Hysteresis loops of Zn_0.4_Fe_2.6_O_4_ NPs acquired at 4 K and 300 K. Inset represents the low-field regime of hysteresis loops. (**e**) Hydrodynamic diameter resulted from DLS measurements of Zn_0.4_Fe_2.6_O_4_ NPs dispersed in water at a concentration of 10 μg_MNPs_/mL.

It is well known that non-stoichiometric Zn ferrite NPs (Zn_x_Fe_3-x_O_4_), with a Zn content in the range 0.3 < x < 0.5 and exhibiting a cubic shape represent very efficient nano heaters for magnetic hyperthermia. For that reason, this class of MNPs was chosen as the magnetic core for this study. The Zn ferrite NPs have been synthesized following the thermal decomposition method elaborated by Noh et al. [45]. As revealed by TEM (Figure 1a), the obtained Zn ferrite NPs have a mean edge length of 28 ± 2 nm, and they are approximatively cubic-like in shape. The Fe and the Zn elements are homogeneously distributed within the total volume of the MNPs, as shown by energy-dispersive X-ray (EDX) maps of Fe, Zn, and O elements in the Zn ferrite NPs (Figure 1b). No core/shell structures of MNPs containing only Fe or Zn were observed. The quantitative analysis of the EDX spectra recorded over many MNPs provides a mean value of the Zn atomic percentage (x) of approximately 0.4, resulting from the Zn doped iron oxide NPs with the formula Zn_0.4_Fe_2.6_O_4_. The XRD pattern (Figure 1c) of uncoated Zn_0.4_Fe_2.6_O_4_ NPs corresponds to the cubic spinel crystal structure of magnetite. All the characteristic diffraction peaks are slightly shifted to lower 2 θ angles due to the incorporated Zn ions. Furthermore, no peaks related to ZnO are detected, indicating the successful incorporation of Zn into the magnetite lattice and the formation of spinel Zn_0.4_Fe_2.6_O_4_. The corresponding lattice parameter was found to be a = 0.8410(4) nm, significantly higher than that of magnetite (a = 0.8396 nm), indicating that the Zn ions occupy the tetrahedral sites. The average crystalline size (27 nm), calculated using Scherrer’s formula by Gaussian fit of the peaks (220), (311), and (440), matches the average edge length obtained from TEM images, suggesting that most of Zn_0.4_Fe_2.6_O_4_ are single crystals. The magnetic characterization of the uncoated Zn_0.4_Fe_2.6_O_4_ MNPs revealed the preferential incorporation of Zn ions at the tetrahedral sites, thus leading to a significant increase in the saturation magnetization (M_s_) to 100 emu/g with respect to that of bulk magnetite (90 emu/g), as indicated by the low temperature (4 K) hysteresis loop (Figure 1d). The M_s_ decreases to 72 emu/g at room temperature, which is consistent with the literature [23,28,46,47], and it can be attributed to increased spin-disorders in the surface layers of smaller MNPs [48]. The ferrimagnetic character of Zn_0.4_Fe_2.6_O_4_ is preserved at room temperature, as can be seen from the inset of Figure 1d; the coercive field (H_c_) slightly decreases from 29 mT (24 kA/m) to 19 mT (15 kA/m) by increasing the temperature from 4 to 300 K. Upon water transfer through the oxidation of oleic acid by sodium periodate, the Zn_0.4_Fe_2.6_O_4_ acquired a zeta potential of −52 mV due to the resulted carboxyl groups [49], indicating good colloidal stability. However, according to DLS data, the ferrimagnetic Zn_0.4_Fe_2.6_O_4_ NPs have a mean hydrodynamic diameter of 70 nm in water, signifying that they stabilize in an aqueous solution in very small clusters, comprising only a few NPs (Figure 1e).

### 3.2. Silica Coating of Magnetic Nanoparticles

**Figure 2:**
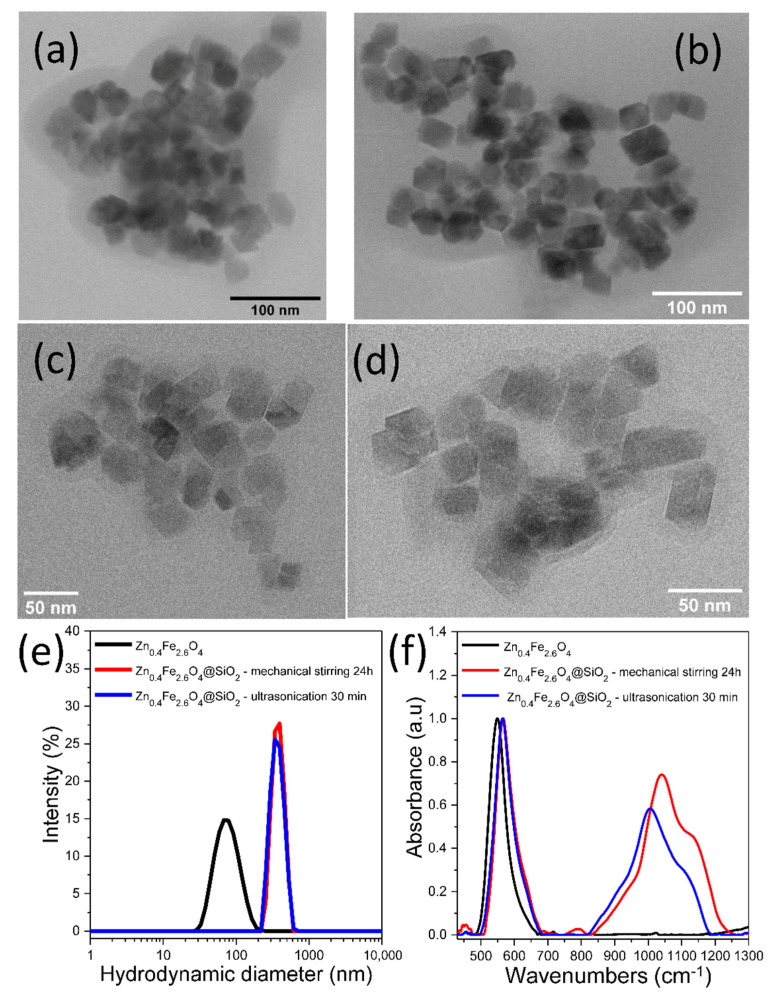
TEM images of Zn_0.4_Fe_2.6_O_4_@SiO_2_-W clusters made via *w*/*o* microemulsion using (**a**,**b**) mechanical stirring for 24 h and (**c**,**d**) ultrasonication for 30 min. (**e**) Hydrodynamic diameter resulted from DLS measurements of uncoated Zn_0.4_Fe_2.6_O_4_ and Zn_0.4_Fe_2.6_O_4_@SiO_2_-W clusters dispersed in water at a concentration of 10 μg_MNPs_/mL. (**f**) FT-IR spectra of uncoated Zn_0.4_Fe_2.6_O_4_ and Zn_0.4_Fe_2.6_O_4_@SiO_2_-W clusters. The spectra are normalized to the highest absorption band.

The classical coating method consisting of mechanical stirring over 24 h of a *w*/*o* (reverse) microemulsion has been firstly applied to encapsulate the hydrophobic Zn_0.4_Fe_2.6_O_4_ clusters in a silica shell. This sample also represents a reference for the present study. As seen in the TEM images of Figure 2a,b, clusters of several tens of MNPs are encapsulated in a thin silica shell and denoted Zn_0.4_Fe_2.6_O_4_@SiO_2_-W. The mean hydrodynamic diameter of the resulted Zn_0.4_Fe_2.6_O_4_@SiO_2_ clusters increased considerably to 400 nm with respect to that of uncoated Zn_0.4_Fe_2.6_O_4_ NPs (Figure 2e). As evidenced by FT-IR spectroscopy, the absorption band originated from the Fe-O bond dominates the spectra, and it is slightly deviated to 566 cm^−1^ (compared to 549 cm^−1^ for uncoated Zn_0.4_Fe_2.6_O_4_) due to the attachment of SiO_2_ to the NP’s surface (Figure 2f). The wide absorption band located at 1039 cm^−1^, due to the stretching vibration of the Si-O-Si bond, proves the presence of the thin layer of SiO_2_ around the Zn_0.4_Fe_2.6_O_4_ clusters (red curve in Figure 2f) [37].

In the second phase of our study, the mechanical stirring of the *w*/*o* microemulsion was replaced by ultrasonication [50]. The experiments revealed that 30 min ultrasonication of the same reaction composition resulted in the formation of a silica layer around Zn_0.4_Fe_2.6_O_4_ clusters (Figure 2c,d). The Zn_0.4_Fe_2.6_O_4_@SiO_2_-W clusters displayed a similar mean hydrodynamic diameter as those formed by mechanical stirring (Figure 2e). The silica layer is slightly thinner as evidenced by the deviation of the Fe-O bond absorption band to 564 cm^−1^ and the occurrence of a less intense Si-O-Si bond absorption band located at 1005 cm^−1^ (blue curve in Figure 2f). Therefore, the ultrasonication considerably reduces the actual formation time of the SiO_2_ layer around Zn_0.4_Fe_2.6_O_4_ clusters through the *w*/*o* microemulsion method from 24 h to 30 min. It has to be mentioned that mechanical stirring allows SPIONs to remain colloidally distributed throughout the mixture volume during the entire silanization process, but in the case of larger or F-MNPs stabilized in clusters, there is a risk of sedimentation. The high energy supplied to the mixture by ultrasounds led primarily to fine emulsification of the internal phase. In other words, very small water droplets were formed, producing a significant increase in the surface area available for the hydrolysis of SiO_2_ precursors at the interface between the two phases. At the same time, the colloidal stability of the dispersion was maintained, and when F-MNPs are used, the ultrasounds prevented their agglomeration. The ultrasounds also increased the number of collisions between Zn_0.4_Fe_2.6_O_4_ clusters, dispersed in the external phase of the mixture, and the water droplets containing ammonia (internal phase of the mixture). Simultaneously with these three actions, the energy released by ultrasounds accelerated the hydrolysis of SiO_2_ precursors at the interface between the two phases, leading to the formation of the SiO_2_ layer within 30 min.

**Figure 3:**
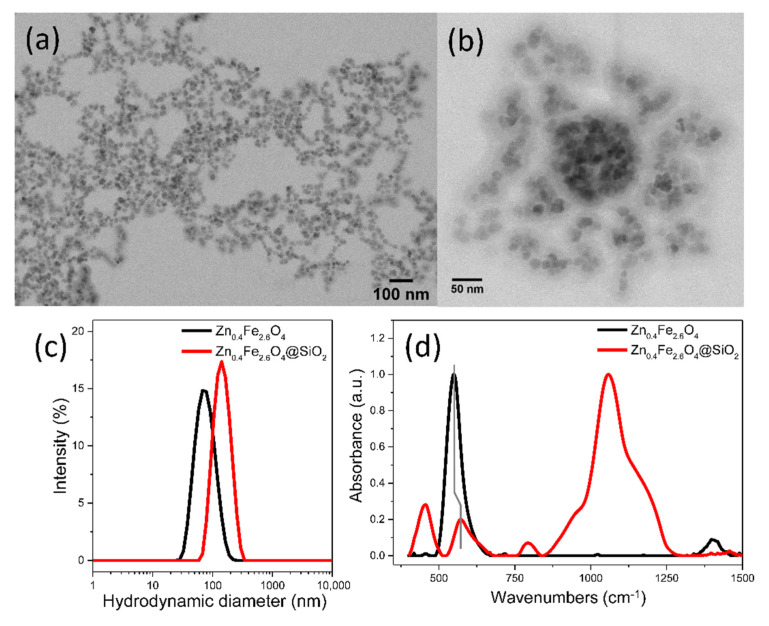
(**a**) Large scale and (**b**) zoom-in TEM images of Zn_0.4_Fe_2.6_O_4_@SiO_2_-O clusters. (**c**) Hydrodynamic diameter resulted from DLS measurements of Zn_0.4_Fe_2.6_O_4_ and Zn_0.4_Fe_2.6_O_4_@SiO_2_-O clusters dispersed in water at a concentration of 10 μg_MNPs_/mL. (**d**) FT-IR spectra of Zn_0.4_Fe_2.6_O_4_ and Zn_0.4_Fe_2.6_O_4_@SiO_2_-O clusters. The spectra are normalized to the highest absorption band.

The significant reduction of the silica coating time to 30 min facilitates the preparation in a short time of large quantities necessary for both in vivo and in vitro evaluation. However, the high average hydrodynamic diameter of Zn_0.4_Fe_2.6_O_4_@SiO_2_-W clusters of 400 nm might represent a disadvantage for such studies. To decrease the average hydrodynamic diameter of Zn_0.4_Fe_2.6_O_4_@SiO_2_-W clusters, different strategies within *w*/*o* microemulsion, consisting of variations in the concentration of internal phase (water droplets) and external phase (MNPs, IGEPAL-CO520, ammonia, or TEOS) have been applied. Unfortunately, all attempts to obtain smaller clusters failed, the only positive result achieved was the control over the SiO_2_ layer thickness of Zn_0.4_Fe_2.6_O_4_@SiO_2_-W clusters. In this regard, the *w*/*o* microemulsion has been replaced with an o/w microemulsion. In this case, the internal phase consists of droplets containing hydrophobic Zn_0.4_Fe_2.6_O_4_ NPs dispersed in hexane, and TWEEN 20 was used as the surfactant. By ultrasonication, these droplets become very small and disperse into the entire volume of the external phase, which is a diluted aqueous solution of ammonia. These very small droplets, containing hydrophobic Zn_0.4_Fe_2.6_O_4_ NPs, practically inhibit their agglomeration into micrometric aggregates, thus offering the possibility to encapsulate the nanometric size clusters of Zn_0.4_Fe_2_._6_O_4_ NPs confined in droplets in an SiO_2_ layer (Figure 3a,b) by the rapid hydrolysis of TEOS at the interface of the two phases under the action hydroxide ions generated by ammonia. According to the DLS measurements, the average hydrodynamic diameter of Zn_0.4_Fe_2.6_O_4_@SiO_2_-O clusters is approximately 145 nm (Figure 3c). As can be seen in TEM images (Figure 3a,b), the resulted SiO_2_ layer is thicker as compared to the previous two samples (Figure 2a–d). In this case, the FTIR spectrum is dominated by the absorption bands characteristic of the SiO_2_ layer (red curve in Figure 3d): the stretching vibration band of the Si-O-Si bond located at 1060 cm^−1^ is the most intense, followed by the bending vibration band of Si-O-Si bond (455 cm^−1^) and the stretching vibration band of the Si-O bond (791 cm^−1^). The shoulder at 965 cm^−1^ is attributed to Si-OH vibrations (Figure 3d). The less intensive absorption band corresponds to the Fe-O bond being noticeable deviated to 573 cm^−1^, as compared to the uncoated sample (Figure 3d).

### 3.3. Magnetic Hyperthermia Capabilities

**Figure 4:**
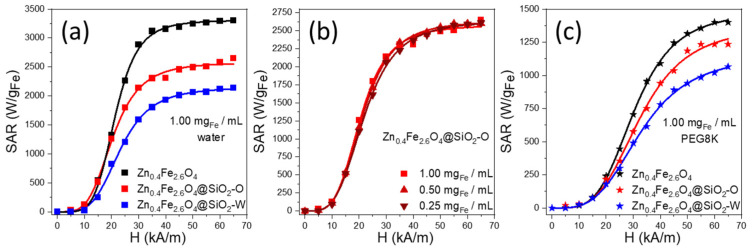
Field dependence of Specific Absorption Rate (SAR) for (**a**) Zn_0.4_Fe_2.6_O_4_, Zn_0.4_Fe_2.6_O_4_@SiO_2_-W, and Zn_0.4_Fe_2.6_O_4_@SiO_2_-O clusters dispersed in water at 1.00 mg_Fe_/mL; (**b**) Zn_0.4_Fe_2.6_O_4_@SiO_2_-O clusters dispersed in water at 1.00, 0.50, and 0.25 mg_Fe_/mL; (**c**) Zn_0.4_Fe_2.6_O_4_, Zn_0.4_Fe_2.6_O_4_@SiO_2_-W, and Zn_0.4_Fe_2.6_O_4_@SiO_2_-O clusters dispersed in PEG8K at 1.00 mg_Fe_/mL. Curves represent the fits with the logistic function (Section S2).

The Specific Absorption Rate (SAR) values of Zn_0.4_Fe_2.6_O_4_@SiO_2_ clusters, formed by *w*/*o* and *o*/*w* microemulsion using the ultrasounds and dispersed either in water or PEG8K, obtained by calorimetric measurements (Figures S1–S3), were measured as a function of the amplitude (H) of the applied alternating magnetic field (AMF) at a fixed frequency (355 kHz), as shown in Figure 4. The SAR evolution with H presents a sigmoidal shape, which is characteristic of F-MNPs with a nonzero hysteresis at the measuring temperature. In the absence of any analytical expression, the SAR evolution with H can be fitted using a simple logistic function (Section S1). This type of function allows the calculation of the most important parameters for characterizing the heating performances of MNPs such as the SAR_max_ (the saturation value of SAR), the H_chyp_ (the hyperthermia coercive field, and the point of maximum slope in the SAR = f(H) function). At a concentration of 1 mg_Fe_/mL, the SAR values of uncoated Zn_0.4_Fe_2.6_O_4_ clusters are 20 and 105 W/g_Fe_ for 5 and 10 kA/m, respectively. For H of 15 kA/m, which represents the H_c_ at room temperature, the SAR increases four times up to 535 W/g_Fe_, and it continues to rise to 3120 W/g_Fe_ for 35 kA/m. By further increasing the H (40–65 kA/m), the SAR values saturate (SAR_max_) around 3305 W/g_Fe_ (Table S1). In the H range of 5–20 kA/m, the SAR values of Zn_0.4_Fe_2.6_O_4_@SiO_2_-O clusters are similar to those reported for uncoated Zn_0.4_Fe_2.6_O_4_ NPs (Figure 4a). Starting with H of 25 kA/m, the Zn_0.4_Fe_2.6_O_4_@SiO_2_-O clusters exhibit lower SAR values compared to uncoated counterparts, the SAR_max_ reaching 2600 W/g_Fe_ (Table S1). In the H range of 25–65 kA/m, the average difference between MH performances of uncoated and silica-coated Zn_0.4_Fe_2.6_O_4_ clusters is 750 W/g_Fe_. This big difference can be explained by the potential of uncoated Zn_0.4_Fe_2.6_O_4_ NPs to organize in chains under the influence of the AMF. This type of organization increases the magnetic anisotropy of the assembly as compared to individual NPs, and it ultimately leads to an enhancement of the heating performances, as was previously reported for magnetite NPs with comparable sizes and magnetic properties [51]. On the contrary, the silica layer prevents the Zn_0.4_Fe_2.6_O_4_ NPs within clusters from entering into physical contact and associating in long chains along the AMF lines. Alternatively, the silica coating increases the hydrodynamic diameters of clusters, reducing their Brownian motion and consequently, the SAR diminishes. However, a closer look at the TEM images in Figure 3a,b reveals that the Zn_0.4_Fe_2.6_O_4_@SiO_2_-O clusters are not spherical but rather elongated. The individual Zn_0.4_Fe_2.6_O_4_ NPs inside these silica-coated clusters seem to be assembled in small chains (resembling magnetosomes) [52], a configuration that facilitates a good MH performance of Zn_0.4_Fe_2.6_O_4_@SiO_2_-O clusters. The SAR values reported for Zn_0.4_Fe_2.6_O_4_@SiO_2_-O clusters are superior as compared to individual Zn_0.4_Fe_2.6_O_4_@SiO_2_ NPs (15.39 kA/m, 525 kHz) [28] or Zn_0.5_Fe_2.5_O_4_@SiO_2_ NPs (5–30 kA/m, 430 kHz) [20]. On the other hand, for H of 15 kA/m (380 kHz), less coercive Zn_0.3_Fe_2.7_O_4_ ferrites, individually coated in the SiO_2_ layer, exhibit double SAR value (1010 W/g_Fe_) [23]. Nevertheless, by increasing the H, the difference between SAR values is reduced, while for H of 35 kA/m the SAR values are almost identical [23].

Over the entire H range, the Zn_0.4_Fe_2.6_O_4_@SiO_2_-W clusters exhibit lower SAR values with respect to Zn_0.4_Fe_2.6_O_4_@SiO_2_-O clusters. The hyperthermia coercive field (H_cHyp_) increased to 20.6 kA/m, while the SAR_MAX_ decreased by 500 W/g_Fe_ as compared to Zn_0.4_Fe_2.6_O_4_@SiO_2_-O clusters (Table S1). The distinctive hyperthermia performance of the two types of silica-coated clusters is related to the strength of magnetic dipole–dipole interaction manifested between Zn_0.4_Fe_2.6_O_4_ NPs composing the clusters. Nanoencapsulation of a large number of Zn_0.4_Fe_2.6_O_4_ NPs in a silica layer, as in the case of Zn_0.4_Fe_2.6_O_4_@SiO_2_-W clusters, leads to an increase of dipole–dipole interactions that is detrimental to SAR [10,53]. On the other hand, the small Zn_0.4_Fe_2.6_O_4_@SiO_2_-O clusters, have larger mobility and better colloidal stability under AMF. On the contrary, the big Zn_0.4_Fe_2.6_O_4_@SiO_2_-W clusters are prone to aggregation under the action of AMF as well as to sedimentation at the bottom of the vial, reducing the Brownian movement and hence the heating performance.

Weakly coercive F-MNPs usually exhibit an increase of SAR by decreasing the concentration, as dipole-dipole interactions are minimized [54,55,56]. The situation is the opposite for highly coercive F-MNPs for which the SAR values decrease with decreasing the concentration [57,58,59]. As can be seen in Figure 4b, there is no variation in SAR values with the concentration for Zn_0.4_Fe_2.6_O_4_@SiO_2_-O clusters. The saturation value of SAR (SAR_Max_) and the hyperthermia coercive field (H_cHyp_) remain almost constant when decreasing the concentration from 1 mg_Fe_/mL down to 0.25 mg_Fe_/mL (Table S1). This is not the case for both Zn_0.4_Fe_2.6_O_4_ and Zn_0.4_Fe_2.6_O_4_@SiO_2_-W clusters, where a variation of SAR values with the colloidal concentration has been recorded (data not shown). This experimental evidence suggests that the thick silica layer around Zn0.4Fe2.6O4 clusters inhibits the inherent magnetic dipole–dipole interactions [60] between them that are responsible for the SAR variation with the concentration, making the Zn0.4Fe2.6O4@SiO2-O clusters excellent candidates for in in vivo and in vitro experiments.

The suppression of magnetic dipole–dipole interactions between Zn_0.4_Fe_2.6_O_4_@SiO_2_-O clusters represents an advantage for in vitro MH experiments since this type of interaction in conjunction with reduced mobility of MNPs confined in endosomes inside the cytosol are responsible for their lower heating efficiency in vitro [61]. To obtain a realistic heating performance, the three types of clusters were immobilized in PEG8K. As plotted in Figure 4c, the SAR values of all three samples are reduced in comparison with water (Figure 4a). In general, the SAR reduction is 80–85% for H of 15 kA/m, and it diminishes to 50–55% by increasing H to 65 kA/m. On average, over the entire H range, a reduction of SAR by 68%, 65%, and 62% for Zn_0.4_Fe_2.6_O_4_ NPs, Zn_0.4_Fe_2.6_O_4_@SiO_2_-O clusters, and Zn_0.4_Fe_2.6_O_4_@SiO_2_-W clusters, respectively, were recorded. These observations suggest that the Brownian relaxation mechanism is the main heating mechanism of both types of silica-coated clusters (in the liquid phase), as demonstrated for the mesoscale assemblage of iron oxide nanocubes [53]. Similar to the aqueous medium, the SAR values in PEG8K of both Zn_0.4_Fe_2.6_O_4_ and Zn_0.4_Fe_2.6_O_4_@SiO_2_-O clusters are identical up to H of 20 kA/m. From 25 kA/m to 65 kA/m, a small average difference of 150 W/g_Fe_ is observed in favor of uncoated Zn_0.4_Fe_2.6_O_4_ clusters. The immobilization of uncoated Zn_0.4_Fe_2.6_O_4_ NPs in PEG restricts their association in chains, and hence reduces the difference between their MH performances and those of Zn_0.4_Fe_2.6_O_4_@SiO_2_-O clusters by a factor of five. Since chain formation is hindered by the silica layer, comparing the MH performances of two types of silica-coated clusters showed that the average difference of SAR values decreased only by a factor of three, from 480 W/g_Fe_ in water to 160 W/g_Fe_ in PEG8K. As observed from Figure 4c, the average value of SAR, in PEG 8 K and on H ranging from 25 to 65 kA/m, decreases progressively by 150–160 W/g_Fe_, when passing from uncoated Zn_0.4_Fe_2.6_O_4_ NPs to small Zn_0.4_Fe_2.6_O_4_@SiO_2_-O clusters and finally to big Zn_0.4_Fe_2.6_O_4_@SiO_2_-W clusters. Since the Brownian motion is suppressed in the solid matrix (PEG8K), the progressive reduction of SAR values can be mainly attributed to the increased strength of magnetic dipole–dipole interactions manifested between Zn_0.4_Fe_2.6_O_4_ NPs within the three types of clusters. Since the Zn_0.4_Fe_2.6_O_4_@SiO_2_-O clusters exhibit the best MF performance among the two types of silica-coated clusters, they were further tested in vitro on a cancer cell line in conjunction with uncoated Zn_0.4_Fe_2.6_O_4_ clusters.

### 3.4. Cellular Internalization and Cytotoxicity

**Figure 5:**
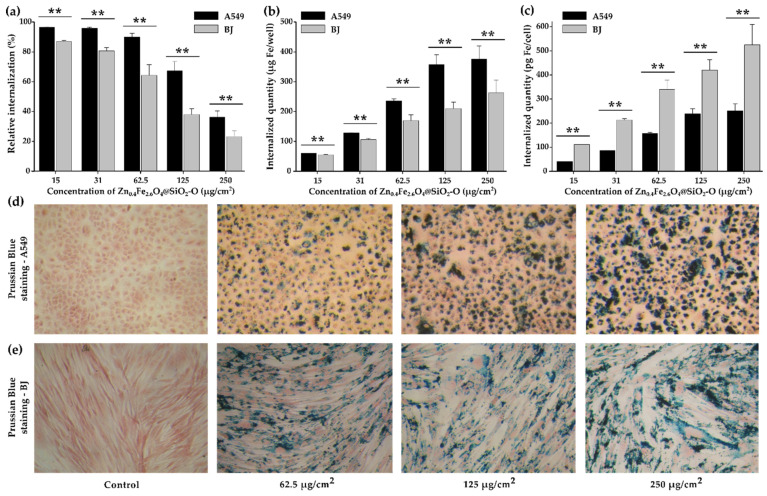
Cellular internalization of Zn_0.4_Fe_2.6_O_4_@SiO_2_-O clusters in A549 and BJ cells after a 24 h exposure: (**a**) the relative internalization and (**b**) total iron amount per well and per cell. (**c**) total iron amount per cell. Prussian Blue staining of (**d**) A549 and (**e**) cells exposed for 24 h to Zn_0.4_Fe_2.6_O_4_@SiO_2_-O clusters. Values are expressed as mean ± SD of three biological replicates. Double asterisks (**) indicate a significant difference with *p* values < 0.001 (ANOVA + Dunn’s).

The reaction of digestion-free ferric ions with thiocyanate was used to quantitatively evaluate the internalization of both Zn_0.4_Fe_2.6_O_4_ NPs and Zn_0.4_Fe_2.6_O_4_@SiO_2-_O clusters on the A549 and BJ cells. The relative internalization as a function of dosage level (Figure 5a) showed that almost all Zn_0.4_Fe_2.6_O_4_@SiO_2_-O clusters were internalized for the first three doses (15, 31, and 62.5 µg/cm^2^). As indicated in Figure 5b, the iron amount increased twice (60 to 128 µg/well) and then four times (60 to 236 µg/well), as the exposure dose was increased from 15 µg/cm^2^ to 31 µg/cm^2^ and 62.5 µg/cm^2^, respectively. This is translated into a linear dependence of the total iron amount internalized in cells with the dosage level (Figure 5c). By further increasing the exposure dose to 125 µg/cm^2^, a considerable drop of 35% in the relative internalization was recorded (Figure 5a). At the highest tested dose (250 µg/cm^2^), the relative internalization continued to decrease, going below 40%. The iron amount internalized in cells deviated from the linear dose dependence and saturated around 375 µg_Fe_/well (310 pg_Fe_/cell) (Figure 5c). Compared with silica-coated iron oxide clusters that we previously investigated [41], a higher relative internalization was achieved for the Zn_0.4_Fe_2.6_O_4_@SiO_2_-O clusters. Congruent results were reported for SPIONs modified with non-porous and mesoporous silica [62]. The exposure of A549 cells to a dose equivalent to 6.25 µg/cm^2^ resulted in a cellular Fe content ranging from 20–25 pg_Fe_/cell [62], which would be similar to the present study (20 pg_Fe_/cell at a dose of 62.5 µg/cm^2^ taking into account a relative internalization > 95%). In comparison with single-core containing Zn_x_Fe_3-x_O_4_@SiO_2_ NPs, where an Fe content of approximately 60 pg_Fe_/cell was observed after a 12 h incubation with an equivalent dose of 600 µg/cm^2^, the Zn_0.4_Fe_2.6_O_4_@SiO_2_-O clusters exhibited a much higher cellular internalization [20]. Compared with silica-coated iron oxide clusters that we previously investigated [41], a slightly higher relative internalization was achieved for the Zn_0.4_Fe_2.6_O_4_@SiO_2_-O clusters for doses from 15 to 62.5 µg/cm^2^. Independently of the dose used, the BJ cells display a statistically lower cellular internalization (Figure 5a,b). While for the first three doses (15, 31, and 62.5 µg/cm^2^) the relative internalization of Zn_0.4_Fe_2.6_O_4_@SiO_2_-O clusters in A549 cells is close to 100%, the relative internalization started at 86% and slightly decreased to 64% in the case of BJ cells. A high drop in the relative internalization (38%) is recorded for a dosage level of 125 µg/cm^2^, while continuing to diminish to 23% for the highest dose (250 µg/cm^2^). No saturation effect has been observed for BJ cells, the internalized iron amount per well followed a progressively increased from 60 to 255 µg/well by increasing the dose (Figure 5b). Due to a higher cytoplasmatic volume of BJ cells, the cell plate accommodates lower cells number as compared to A549 cells. In this condition, the internalized amount per BJ cell was approximately double (Figure 5c).

The relative internalization for uncoated Zn_0.4_Fe_2.6_O_4_ clusters is only 59% for the smallest dose of 15 µg/cm^2^, and it decreases to 11% for the highest dose of 125 µg/cm^2^ (Figure S5). For the BJ cells, the relative internalization is even lower, decreasing from 25% to 5% over the dose range (Figure S5a). In terms of the internalized iron amount per well, a constant value at approximately 21 µg/well for the first three doses and increasing up to 73 µg/well for the highest dose (Figure S5b) is observed for BJ cells. In the case of A549 cells, the iron amount internalized per well is much higher, ranging from 50 to 123 µg/well over the dose range (Figure S5b). However, when counting the internalized iron amount per cell, there is no difference between the two cell lines for the first three doses (Figure S5c). From a dosage level of 125 µg/cm^2^, the BJ cells accommodate more uncoated Zn_0.4_Fe_2.6_O_4_ clusters than the A549 cells (Figure S5c). All these observations clearly point out that the coating with silica increased the cellular internalization of the Zn_0.4_Fe_2.6_O_4_ NPs by a factor of 1.7 to 3, as the dosage level is increased from 15 to 125 µg/cm^2^. Following nanostructures adsorption on the cellular membrane, internalization occurs through a variety of processes, including pinocytosis, non-specific or receptor-mediated endocytosis, and phagocytosis [63], while the dimension of the nanostructure represents a key factor in cellular internalization. In addition, the Zn_0.4_Fe_2.6_O_4_@SiO_2_-O clusters exhibit a less negative zeta potential (−27 mV) than uncoated Zn_0.4_Fe_2.6_O_4_ clusters (−52 mV). The lower zeta potential could facilitate the adsorption of the Zn_0.4_Fe_2.6_O_4_@SiO_2_-O clusters to the A549 cell membranes as they display extensive negative charged domains that electrostatically repel highly negatively charged NPs [64,65,66].

The Zn_0.4_Fe_2.6_O_4_@SiO_2_-O clusters were indeed effectively internalized by A549 and BJ cells, as revealed by the light microscopic images where agglomerates of Zn_0.4_Fe_2.6_O_4_@SiO_2_-O clusters are visible in the cytoplasm (Figure 5d,e). Regardless of the dosage level of Zn_0.4_Fe_2.6_O_4_@SiO_2_-O clusters, the A549 cells exhibited typical morphology with no shrinkage of the cellular volume, indicative of a cytotoxic effect (Figure 5d,e). A dose-dependent internalization process can be observed by a closer inspection of images. Moreover, a perinuclear deposition of the nanomaterials was observed for both types of cells, with almost no particles being observed in the nuclear area. At the same time, it can be observed that the cytoplasmatic loading with Zn_0.4_Fe_2.6_O_4_@SiO_2_-O clusters was not uniformly realized. A similar observation can be drawn for uncoated Zn_0.4_Fe_2.6_O_4_ when inspecting their corresponding images presented in Figure S5d,e. In addition, the poor internalization of uncoated Zn_0.4_Fe_2.6_O_4_ NPs with respect to Zn_0.4_Fe_2.6_O_4_@SiO_2_-O clusters can be also identified at each dosage level.

**Figure 6:**
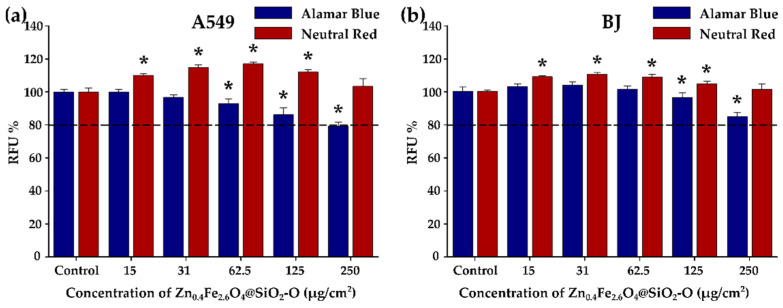
Cytocompatibility of Zn_0.4_Fe_2.6_O_4_@SiO_2_-O clusters on (**a**) A549 and (**b**) BJ cell line after a 24 h exposure. Data are presented as relative fluorescence units (RFU) as compared to the negative control (100%), as mean ± SD of three biological replicates. The significant differences compared to the negative control (ANOVA + Dunn’s; *p* < 0.05) are noted with asterisks (*).

The viability of A549 cancer and BJ normal cells was determined by Alamar Blue (AB) and Neutral Red (NR) assays after incubation for 24 h with various concentrations of Zn_0.4_Fe_2.6_O_4_@SiO_2_-O clusters (Figure 6a,b) and of uncoated Zn_0.4_Fe_2.6_O_4_ clusters (Figure S6a,b) ranging from 15 to 250 µg/cm^2^ (50–800 µg/mL). Cells that were not exposed to Zn_0.4_Fe_2.6_O_4_@SiO_2_-O clusters were used as a negative control. Similar to our previously published studies, the optical and the biochemical interferences were firstly evaluated to avoid artefactual data caused by the interferences of Zn_0.4_Fe_2.6_O_4_@SiO_2_-O clusters with the biochemical assays used [41,59,67]. At a dosage level of 15 µg/cm^2^, the AB assay did not exhibit statistically relevant cytotoxicity (Figure 6a). A slight statistical decrease in viability is recorded by increasing the dosage level (Figure 6a). Even though for doses from 31 to 125 µg/cm^2^ the viability of cells is slightly lower than that of the control group, it is still well above 80%, which is considered the viability threshold for nanomaterials to be safe for biomedical applications [68]. For 250 µg/cm^2^, the Zn_0.4_Fe_2.6_O_4_@SiO_2_ clusters exhibited relevant cytotoxicity, decreasing cellular viability by more than 20% (Figure 6a). Instead, at this dosage level, the viability of BJ cells was still slightly above 80% (Figure 6b). Taking into account that the BJ cells accommodate a double number of silica-coated clusters, the toxicity level of these nanostructures toward normal cells is much lower compared to A549 cells. Conversely, the NR assay displayed an increase in cellular viability starting with the lowest dose (Figure 6a,b). The highest increase in cellular viability was observed at intermediary doses, which did not induce a cytotoxic effect based on the AB assay (Figure 6a,b). At the highest tested dose of 250 µg/cm^2^, the viability was not different from that observed for the negative control for both cell lines. As observed by our group and others, these slight increases in viability measured with the NR assay are most probably due to the increase in the lysosomal compartment that favors the ATP-dependent incorporation of the NR dye in the cells [69,70]. Similarly, an increase in cellular viability based on the NR data and a decrease in viability according to the WST assay was reported by another group for magnetite NPs [70]. The Zn_0.4_Fe_2.6_O_4_@SiO_2_-O clusters are less toxic, compared to silica-coated iron oxide clusters of bigger dimensions (threshold dose of 125 µg/cm^2^) [41], and they exhibit higher toxicity with respect to the single-core containing Zn_x_Fe_3-x_O_4_@SiO_2_ NPs, which showed biocompatibility up to 1000 µg/mL [16,20,23]. Due to the pure internalization of uncoated Zn_x_Fe_3-x_O_4_ clusters in both types of cells, according to the AB assay, no cytotoxicity effect was recorded over the entire dose interval (Figure S6a,b). Similar to the case of Zn_0.4_Fe_2.6_O_4_@SiO_2_-O clusters, the NR assay indicated an increase in cellular viability mainly at intermediate doses (Figure S6a,b).

### 3.5. In Vitro Magnetic Hyperthermia

**Figure 7:**
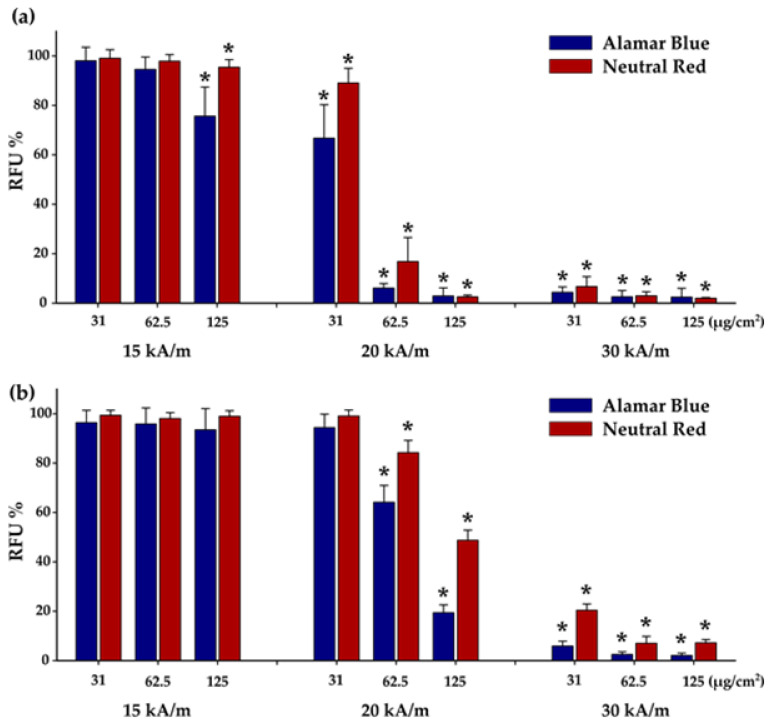
Cytotoxic effects of internalized Zn_0.4_Fe_2.6_O_4_@SiO_2_-O clusters in (**a**) A549 and (**b**) BJ cells were evaluated after a 30 min exposure to AMF of 355 kHz and amplitudes of 15, 20, and 30 kA/m. Cellular viability was measured using Alamar Blue and Neutral Red assays and presented as the mean ± SD of three biological replicates. Data are presented as relative values to AMF negative control (100%). The significant differences compared to the negative control (ANOVA + Dunn’s; *p* < 0.05) are marked with asterisks (*).

A549 and BJ cells were incubated with Zn_0.4_Fe_2.6_O_4_@SiO_2_-O clusters in a concentration range from 31 to 125 µg/cm^2^ for 24 h and then exposed for 30 min to an AMF of variable amplitude (15, 20, and 30 kA/m) and a fixed frequency of 355 kHz. The effect of AMF was evaluated by measuring cellular viability with respect to a control sample by performing both AB and NR assays 24 h after MH treatments. The effect of AMF alone was also evaluated by exposing untreated A549 cells, following the same protocol applied to A549 cells containing Zn_0.4_Fe_2.6_O_4_@SiO_2_-O clusters. This latter exposure was accompanied by a modest increase in temperature, not exceeding 0.5 °C, which did not affect cellular viability [41,59]. The heating curves of A549 cells containing internalized Zn_0.4_Fe_2.6_O_4_@SiO_2_-O clusters exhibited a relevant increase in the temperature in the first 5 min followed by the formation of a plateau (Figure S7). The saturation temperature (T_s_) strongly depends on the amount of internalized Zn_0.4_Fe_2.6_O_4_@SiO_2_-O clusters as well as on the amplitude of AMF (Figure S7), and it can be correlated with cellular viability (Figure 7). Both toxicity assays indicated no changes in cellular viability for a dosage level of 31 and 62.5 µg/cm^2^ at H of 15 kA/m (Figure 7a). This is congruent to small T_s_ values of 39.7 and 41.1 °C reached during MH treatment (Figure S7), which are below the 42 °C considered to be the threshold value necessary to initialize apoptosis in cancer cells. For a dose of 125 µg/cm^2^, the T_s_ increased up to 42.7 °C. At this point, the NR assay indicated a minor change in cellular viability, while the AB assay showed that approximately a quarter of the A549 cells were dead (Figure 7a). For normal cells, no cytotoxic effect was observed for all three doses at an H of 15 kA/m (Figure 7b), the recorded T_s_ being well below 42 °C (Figure S7). The effect of MH treatment in killing A549 cells became relevant at H of 20 kA/m, above the coercive field of Zn_0.4_Fe_2.6_O_4_ NPs (15 kA/m). Firstly, for a dose of 31 µg/cm^2^, cellular viability decreased to 67% according to the AB assay, while the NR assay indicated only a 10% drop in cellular viability. The low percentage of damaged cells can be explained by the T_s_ of 43 °C, which is not enough to induce a statistically major decrease in cellular viability upon 24 h from MH treatment. For the next two doses of 62.5 µg/cm^2^ and 125 µg/cm^2^, the AMF exposure at 20 kA/m enabled reaching T_s_ of 45.3 °C and 47.5 °C, respectively. Consequently, the MH treatment-induced cellular death in more than 90% of A549 cells, as independently indicated by both biochemical assays. A cytotoxic effect was also recorded for BJ cells exposed at H of 20 kA/m; however, the percentages of dead cells were smaller (Figure 7b) compared to A549 cells. For the first dose (31 µg/cm^2^), the T_s_ reached a value of 42 °C, which was not enough to produce a cytotoxic effect (Figure 7b). Starting from the intermediary dose of 62.5 µg/cm^2^, AB and NR assays exhibited a 35% and a 15% decrease in viability, respectively (Figure 7b), which indicates a minor cytotoxic effect in accordance with the reached T_s_ of 43.5 °C (Figure S7). The cytotoxic effect was relevant at the highest exposure dose (125 µg/cm^2^), the viability decreasing to 20% and 50% based on the AB and NR data, respectively (Figure 7b). Independent of the cell type and the viability assay used, the MH treatment at H of 30 kA/m enabled reaching T_s_ well above 45 °C (Figure S7). As a consequence, the viabilities indicated a cellular death of more than 90% of cells (Figure 7a,b).

The cellular viability data obtained using both biochemical assays followed a sigmoidal dependence as a function of T_s_ (Figure S8a), which can be fitted by an equation derived from a two-state model of temperature-dependent cell damage [71]. The fitting results indicated that the temperatures at which the A549 cells, exposed for 30 min to MH treatment, received a 50% lethal dose (LD50%) are 43.44 °C and 44.69 °C for the AB and the NR assays, respectively (Figure S8a). These values are very close to those obtained in previous studies [41,67]. A second important parameter resulting from the fits is the temperature width for a given decrease in cell viability. Both biochemical assays revealed a temperature width of 0.75 °C (Figure S8a), which means that the distribution of A549 cells with different responses to MH treatment is narrow. In other words, the 100% decrease in cellular viability occurs in a temperature interval of less than 1 °C, once initiated through the heat generated by the internalized Zn_0.4_Fe_2.6_O_4_@SiO_2_-O clusters. In the case of BJ cells, the AB and the NR assays showed that a 50% lethal dose was reached at a temperature of 44.22 °C and 45.46 °C, respectively, while a complete destruction (100%) can occur in a temperature interval of 1 °C. Consequently, the BJ cells are more resilient to MH treatment as compared to A549 cells using Zn_0.4_Fe_2.6_O_4_@SiO_2_-O clusters as nanoheaters.

## 4. Conclusions

We have developed a new straightforward method for silica coating of pre-existing clusters of ferromagnetic nanoparticles (F-MNPs) formed by cubic Zn ferrite nanoparticles (NPs). Compared to classical methods of silica coating based on the Stöber process or water-in-oil microemulsion, our method is fast, facile, effective, and eco-friendly. The silica (SiO_2_) coating method resides in using an oil-in-water microemulsion and ultrasounds, which allowed us to obtain Zn_0.4_Fe_2.6_O_4_@SiO_2_ clusters in 30 min, displaying a small hydrodynamic diameter of 145 nm. The produced Zn_0.4_Fe_2.6_O_4_@SiO_2_-O clusters exhibit high heating power when exposed to an alternating magnetic field (AMF) of 355 kHz and variable amplitudes. The Specific Absorption Rate (SAR) increased sigmoidally, with the AMF amplitude reaching a saturation value of 2600 W/g_Fe_. The SAR values did not vary with the colloidal concentration (in the range of 0.25–1 mg/mL), proving that the silica coating significantly reduces the magnetostatic interactions among coercive Zn_0.4_Fe_2.6_O_4_ clusters.

Cytotoxicity studies on the A549 cancer cell line using two complementary assays (Alamar Blue and Neutral Red) revealed a good biocompatibility with a drop of only 22% in cellular viability at the highest dose of 250 μg/cm^2^ (0.8 mg/mL) used. Instead, insignificant toxicity for normal BJ cells was recorded. Cellular uptake experiments revealed that Zn_0.4_Fe_2.6_O_4_@SiO_2_-O clusters are internalized in A549 cells in a linear dose-dependent manner and then saturated at higher doses. BJ cells internalized double the amount of Zn_0.4_Fe_2.6_O_4_@SiO_2_-O clusters per cell, however the relative internalization indicated a smaller cellular uptake as compared to A549 cells. Moreover, the cellular uptake is almost three times higher as compared to the uncoated NPs. This higher uptake is translated into a high intracellular magnetic hyperthermia efficiency, our results revealing that more than 90% of A549 cells, incubated at a dose of 62.5 μg/cm^2^, underwent cellular death close to the upper limits of safe AMF field conditions (20 kA/m, 355 kHz). The sigmoidal fitting of cellular viability as a function of saturation temperature revealed that the LD50% in A549 cells was at a mean temperature of 44 °C. The BJ cells were found to be more resilient to MH treatment, the threshold dose being 125 μg/cm^2^ at the same AMF condition (20 kA/m, 355 kHz), while LD50% was initiated at a mean temperature of 45 °C.

The silica coating methodology elaborated herein is not limited to the use of coercive Zn_0.4_Fe_2.6_O_4_ NPs as core constituents, therefore extensions to other types of NPs may yield new hybrid nano-objects. The suppression of strong magnetostatic interactions by the silica layer and the fact that the silica-coated clusters are easy to disperse in water, open up the coercive MNPs for a large range of applications. Moreover, the silica surface of the clusters offers multiple possibilities for further functionalization, either by targeting ligands or by chemotherapeutic drugs opening multiple ways for biomedical applications. The high heating performances of the developed Zn_0.4_Fe_2.6_O_4_ NPs in conjunction with the scalability of the oil-in-water microemulsion silica coating procedure could facilitate a successful implementation of the Zn_0.4_Fe_2.6_O_4_@SiO_2_-O clusters into clinical applications.

## Data Availability

Not applicable.

## Supplementary Materials

The following supporting information can be downloaded at: https://www.mdpi.com/article/10.3390/biomedicines10071647/s1, Figures S1–S3: Heating curves and their corresponding temperature change ΔT versus time curves fitted with Box–Lucas equation; Figure S4: Calibration curve for iron concentration determination; Figure S5: Cellular internalization of uncoated Zn_0.4_Fe_2.6_O_4_ clusters; Figure S6: Cytotoxicity of uncoated Zn_0.4_Fe_2.6_O_4_ clusters; Figure S7: Heating curves of in vivo MH treatment; Figure S8: Cellular viability versus saturation temperature; Table S1: Fitting parameters of SAR evolution with H.
