# Supplementary material for: A Fast, Reliable Oil-In-Water Microemulsion Procedure for Silica Coating of Ferromagnetic Zn Ferrite Nanoparticles Capable of Inducing Cancer Cell Death In Vitro
Source: Biomedicines. 2022 Jul 8;10(7):1647. doi: 10.3390/biomedicines10071647 (PMC9313231; doi:10.3390/biomedicines10071647)
Supplement: Supplementary file 1 [file biomedicines-10-01647-s001.zip › biomedicines-1771237-supplementary.pdf]

Supplementary Materials

Fast and reliable oil-in-water micro-emulsion procedure for silica coating of ferromagnetic Zn ferrite nanoparticles capable to induce cancer cells death *in vitro*

Stefan Nitica[1‡], Ionel Fizesan[2‡], Roxana Dudric[3], Lucian Barbu Tudoran[4,5], Anca Pop[2], Felicia Loghin[2], Nicoleta Vedeanu[1], Mihai Constantin Lucaciu[1]* and Cristian Iacovita[1]

[1] *Department of Pharmaceutical Physics-Biophysics, Faculty of Pharmacy, "Iuliu Hatieganu" University of Medicine and Pharmacy, 6 Pasteur St., 400349 Cluj-Napoca, Romania.*

stefan_nitica@yahoo.com (S.N.); nicoletavedeanu@yahoo.com (N.V.); clucaciu@umfcluj.ro (M. C. L.); cristian.iacovita@umfcluj.ro (C.I.);

[2] *Department of Toxicology, Faculty of Pharmacy, "Iuliu Hațieganu" University of Medicine and Pharmacy, 6A Pasteur St., 400349 Cluj-Napoca, Romania. ionel.fizesan@umfcluj.ro (I.F.); anca.pop@umfcluj.ro (A.P.); floghin@umfcluj.ro (F.L.)*

[3] *Faculty of Physics, "Babes Bolyai" University, Kogalniceanu 1, 400084 Cluj-Napoca, Romania. roxana.dudric@ubbcluj.ro (R.D.)*

[4] *Electron Microscopy Center "Prof. C. Craciun", Faculty of Biology & Geology, "Babes-Bolyai" University, 5-7 Clinicilor St., 400006 Cluj-Napoca, Romania. lucian.barbu@ubbcluj.ro*

[5] *Electron Microscopy Integrated Laboratory, National Institute for Research and Development of Isotopic and Molecular Technologies, 67-103 Donath St., 400293 Cluj-Napoca, Romania.*

* Corresponding author: C.M. Lucaciu clucaciu@umfcluj.ro, 0040-744-647-854

‡These authors contributed equally.

S1. Magnetic hyperthermia

The specific absorption rate (SAR) is defined as the heat released from a suspension of MNPs in unit time reported to the mass of iron content. It was used to quantify the heat performance of MNPs. For reliable determination of SAR, the temperature change ΔT versus time curves - where ΔT = T(t) − T0; T(t) is the temperature at time t and T0 = 37 °C -, have been fitted with the Box-Lucas equation:

$$\Delta T = \frac{S_m}{k}\left(1 - e^{-k(t-t_0)}\right) \qquad (1)$$

where the fitting parameters S_m and k are the initial slope of the heating curve and the constant describing the cooling rate, respectively. Thus, SAR can be calculated as:

$$\mathrm{SAR} = \frac{c\, m\, S_m}{m_{Fe}} \qquad (2)$$

where c is the specific heat of the colloid (in our case was approximated with the specific heat of water: $c = 4186\ J/kgK$ and PEG8K: $c = 2136\ J/kgK$ the MNPs contribution to the specific heat being negligible), $m = \rho/V$ is the mass of colloid, taken as the product between the density ($\rho_{water} = 0,997\ g/cm^3$ for water and $\rho_{PEG8K} = 1,125\ g/cm^3$ at 298K) and the volume. The iron concentration of samples was determined using the thiocyanate assay described in the section below. Prior to each measurement, the samples have been sonicated for 10 seconds to assure a good colloidal dispersion over the entire aqueous volume. Each SAR value is a mean of three measurements realized on three different samples.

The sigmoidal evolution of our experimental SAR data with H was well fitted (R^2 > 0.999) phenomenologically with a simple logistic function:

$$SAR = SAR_{max}\frac{\left(\frac{H}{H_{cHyp}}\right)^{n} * \propto}{1 + \left(\frac{H}{H_{cHyp}}\right)^{n} * \propto} \qquad (3)$$

with:

$$\propto = \frac{n+1}{n-1} \qquad (4)$$

where SAR_{max} - the saturation value of the SAR, H_{cHyp} - the hyperthermia coercive field, the value of the H for which the function presents the highest slope or the H at which the first derivative of SAR against H presents a maximum, and the exponent n – which indicates how steep is the dependence of SAR on H. The values of these parameters for all four types of MNPs at each iron concentration are provided in Table S1 below.

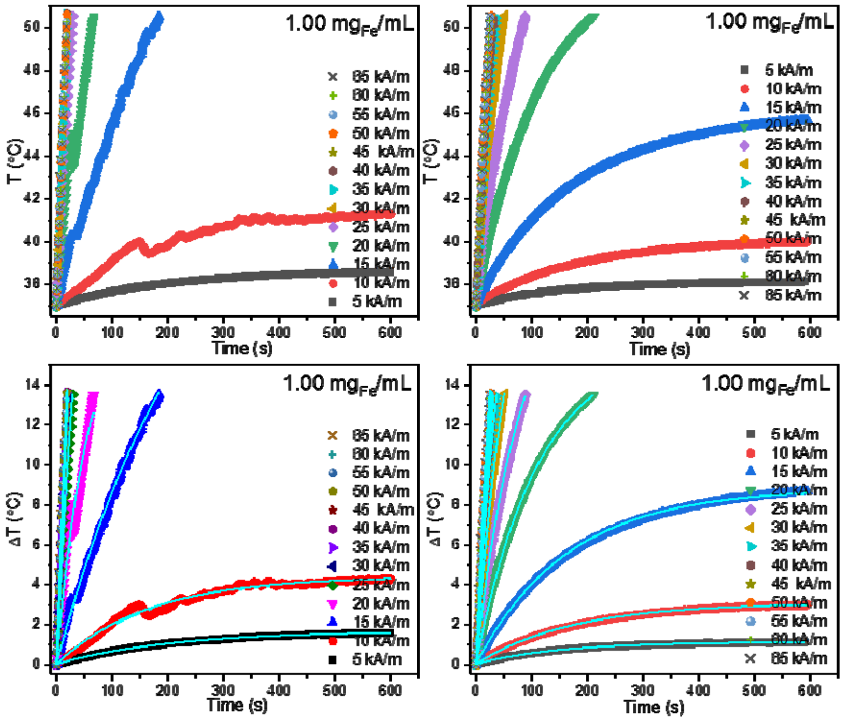

Figure S1. Groups of panels displaying the heating curves and their corresponding temperature change ΔT versus time curves fitted with Box-Lucas equation (blue curves) of $Zn_{0.4}Fe_{2.6}O_4$ NPs, dispersed in water (left panels), and PEG8K (right panels) at an iron concentration of 1.00 mg_{Fe}/mL, recorded as a function of H (5 – 65 kA/m) at a frequency of 355 kHz.

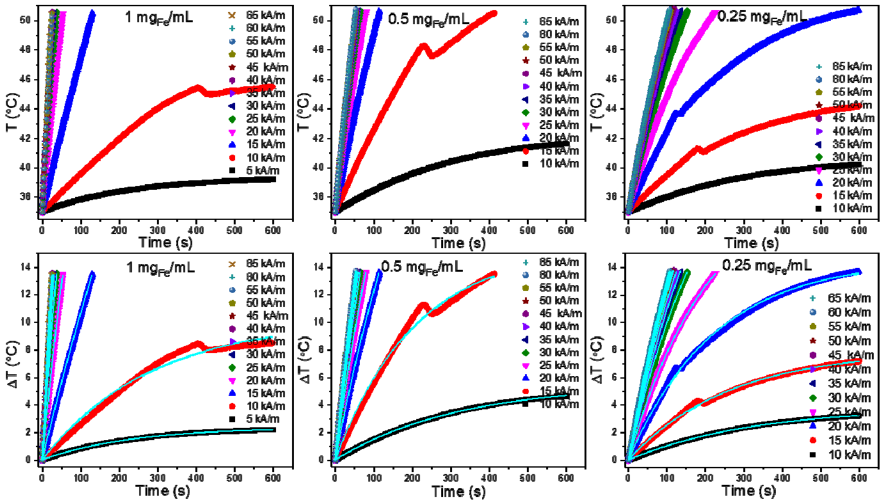

Figure S2. Groups of panels displaying the heating curves and their corresponding temperature change ΔT versus time curves fitted with Box-Lucas equation (blue curves) of $Zn_{0.4}Fe_{2.6}O_4$@SiO_2-O clusters, dispersed in water at an iron concentration of 1.00 mg_{Fe}/mL (left panels), 0.50 mg_{Fe}/mL (middle panels) and 0.25 mg_{Fe}/mL (right panels) recorded as a function of H (5 – 65 kA/m) at a frequency of 355 kHz.

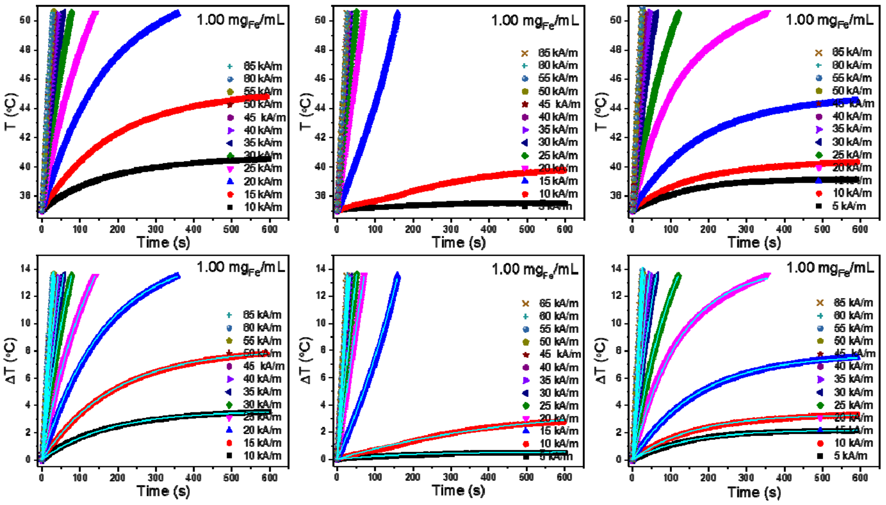

Figure S3. Groups of panels displaying the heating curves and their corresponding temperature change ΔT versus time curves fitted with Box-Lucas equation (blue curves) of $Zn_{0.4}Fe_{2.6}O_4$@SiO_2-O clusters, dispersed in PEG8K at an iron concentration of 1.00 mg_{Fe}/mL (left panels) and $Zn_{0.4}Fe_{2.6}O_4$@SiO_2-W clusters, dispersed in water (middle panels), and PEG8K (right panels) at an iron concentration of 1.00 mg_{Fe}/mL recorded as a function of H (5 – 65 kA/m) at a frequency of 355 kHz.

Table S1. Fitting parameters of SAR evolution with H.

**Sample**	**Conditions**	**c (mg_{Fe}/mL)**	**SAR_{max} (W/g_{Fe})**	**H_{cHyp} (kA/m)**	**Power Coefficient n**
$Zn_{0.4}Fe_{2.6}O_4$	water	1.00	3305±21	19.8±0.2	5.1±0.2
	PEG8K		1480±13	27.0±0.2	4.1±0.1
$Zn_{0.4}Fe_{2.6}O_4$@SiO_2-O	water	1.00	2590±28	18.3±0.3	4.1±0.3
		0.50	2635±13	18.6±0.1	3.9±0.1
		0.25	2640±18	18.9±0.2	3.7±0.1
	PEG8K	1.00	1370±35	28.2±0.7	3.7±0.2
$Zn_{0.4}Fe_{2.6}O_4$@SiO_2-W	Water	1.00	2150±22	20.6±0.3	4.1±0.2
	PEG8K		1155±20	28.2±0.5	3.5±0.1

S2. Iron concentration determination calibration curve

The iron content of $Zn_{0.4}Fe_{2.6}O_4$@SiO_2 NPs was measured using the Liebig reaction. In this regard, approximately 5 mg of $Zn_{0.4}Fe_{2.6}O_4$@SiO_2 NPs were magnetically separated and further mixed with a 10 mL of 12% HCl solution. The digestion was performed for 6 h at 80 °C, and the obtained solutions were centrifuged at 12,000 g for 10 mins to obtain the supernatants. The total Fe^{3+} content of supernatants (50 µL) was measured after an oxidation step with 1% ammonium persulfate (50 µL) by the reaction with 0.1 M potassium thiocyanate (100 µL) that yields a red-colored iron-thiocyanate complex. The absorbance was measured at λ = 490 nm using a Synergy 2 Multi-Mode Microplate Reader, and the Fe^{3+} content was calculated from a standard curve with concentrations ranging between 5 - 140 µg/mL (Fig. S1).

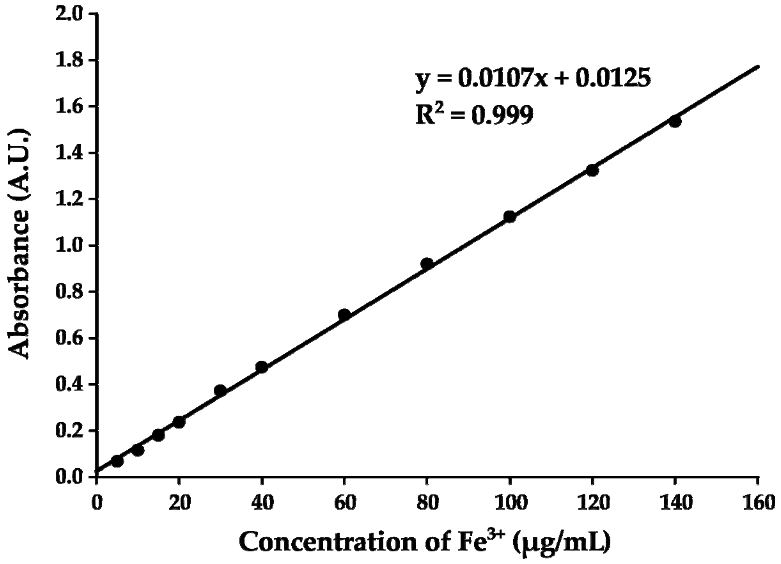

Figure S4. The absorbance of six standard Fe^{3+} colloidal solutions as a function of Fe^{3+} concentration measured at a λ = 490 nm. The values are expressed as the mean ±SD of three replicates. The black line represents a linear regression of the experimental values.

S3. Cellular internalization of uncoated $Zn_{0.4}Fe_{2.6}O_4$ clusters

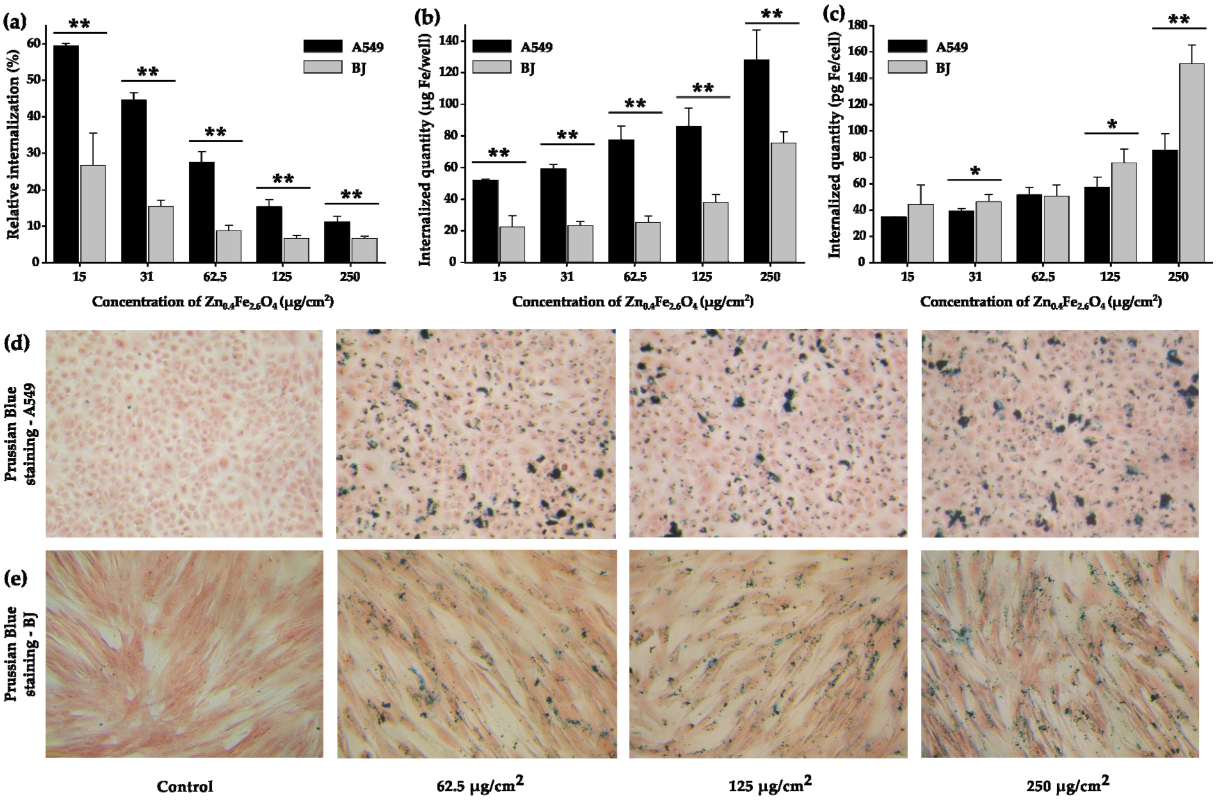

Figure S5. Cellular internalization of uncoated $Zn_{0.4}Fe_{2.6}O_4$ in A549 and BJ cells after a 24 h exposure: (**a**) the relative internalization and (**b**) total iron amount per well and (**c**) total iron amount per cell. Prussian Blue staining of (**d**) A549 and (**e**) BJ cells exposed for 24 h to $Zn_{0.4}Fe_{2.6}O_4$@SiO_2-O clusters. Values are expressed as mean ± SD of three biological replicates. Asterisks (*) indicate significant differences compared to the negative control (ANOVA + Dunn’s; $p < 0.05$), and double asterisks (**) indicate a significant difference with a p value <0.001 (ANOVA + Dunn‘s).

S4. Cytotoxicity of uncoated $Zn_{0.4}Fe_{2.6}O_4$ clusters

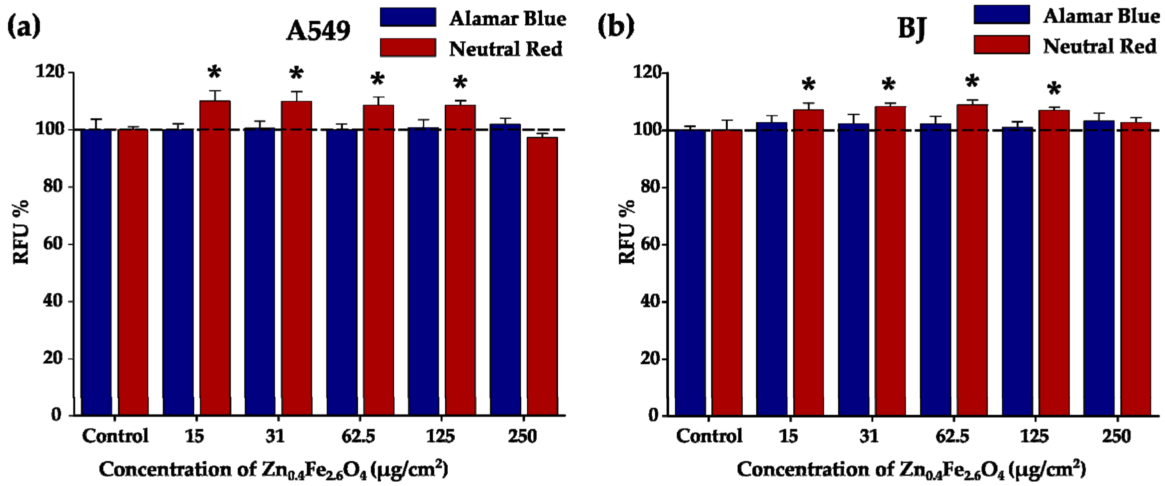

Figure S6. Cytocompatibility of uncoated $Zn_{0.4}Fe_{2.6}O_4$ clusters on (**a**) A549 and (**b**) BJ cell line after a 24 h exposure. Data are presented as relative values to the negative control (100%), as mean ± SD of three biological replicates. The significant differences compared to the negative control (ANOVA + Dunn's; $p < 0.05$) are noted with asterisks (*).

S5. Heating curves of in vivo MH treatment

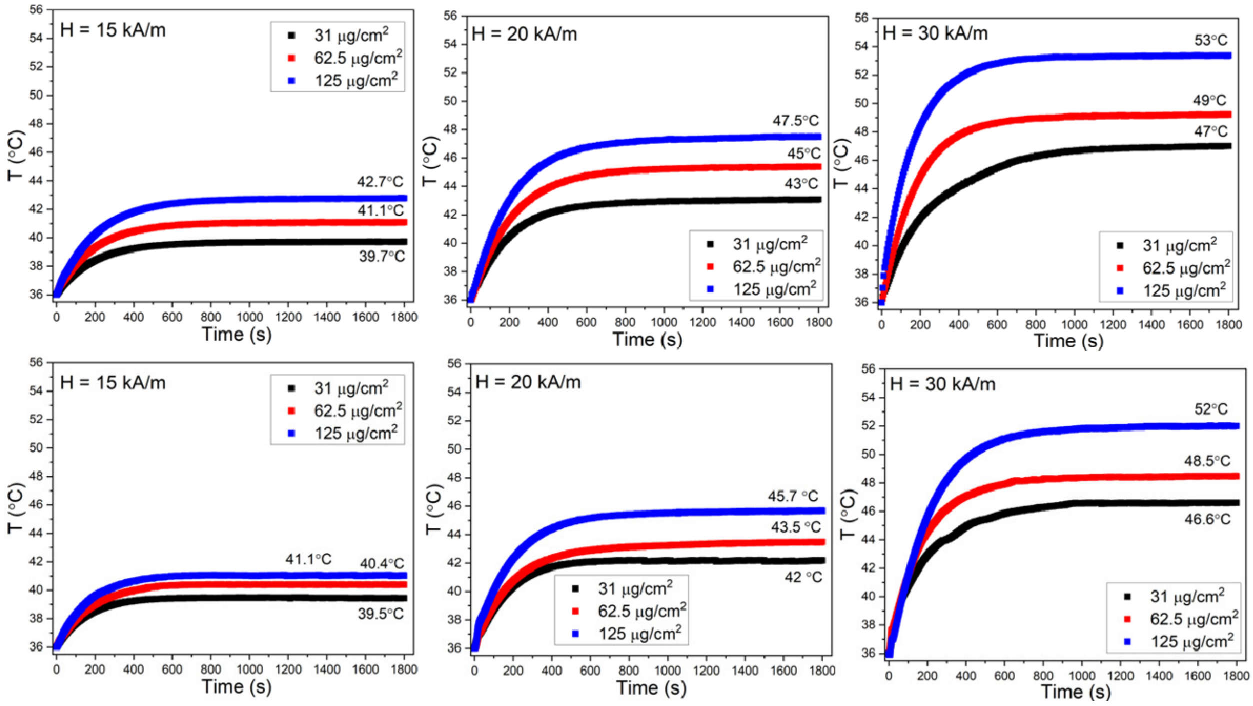

Figure S7. Heating curves of $Zn_{0.4}Fe_{2.6}O_4$@SiO_2-O clusters internalized in A549 (upper panels) and BJ (lower panel) cells at different dosage levels in a volume of 200 µL at different H values: 15 kA/m (left panels), 20 kA/m (middle panels) and 30 kA/m (right panels) at a constant frequency of 355 kHz.

S6. Cellular viability versus saturation temperature

The cellular viability, obtained using both biochemical assays, as a function of saturation temperature, reached during MH treatment, can be fitted with the following sigmoidal function:

$$C(T) = \frac{A}{1 + e^{\frac{T - T_0}{dT}}} \qquad (5)$$

where A represents the viability of control cells (100%) and dT quantifies the temperature width for a given decrease in cell viability, while T_0 represents the temperature at which the viability reaches a value of 50%.

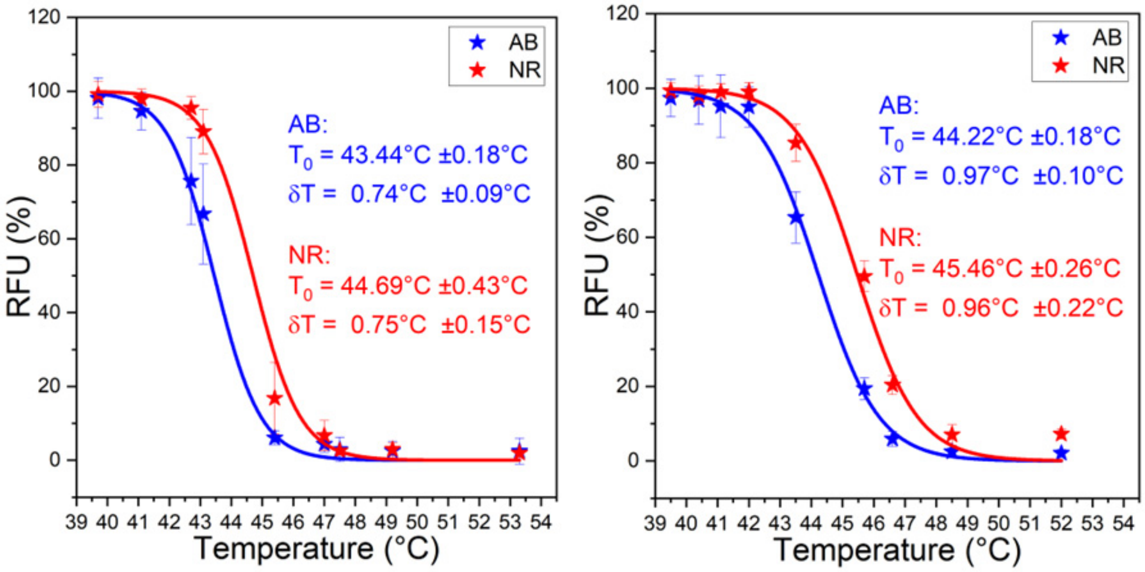

Figure S8. Cellular viability for A549 (left) and BJ (right) cells for $Zn_{0.4}Fe_{2.6}O_4$@SiO_2-O clusters based on AB and NR assays, plotted against the saturation temperatures reached during MH treatment and their corresponding fitting curves and fitting parameters based on equation 5.
